# Abscess of the Lung, Probably Caused by the Stump of a Tooth Passing into Right Bronchus

**Published:** 1888-02

**Authors:** 


### SELECTIONS.

### Abscess of the Lung, Probably Caused by the Stump of a Tooth Passing into Right Bronchus.

Dr. William Stranger, senior physician of the Worcester General Infirmary, reports the following interesting case in the *Brit. Med. Journal*, November 26, 1887:

E. M., a robust young woman, 23 years old, with well-developed chest and fine physique, and in good health, although in infancy she had been the subject of scrofulous abscesses of the hands and feet, took chloroform, for the extraction of several stumps of teeth, on February 17, 1887. A few days afterwards a troublesome irritative cough came on, with slight muco-purulent expectoration. This condition continued until the month of June —four months. During this time she was never entirely laid up, although frequently confined to her bed for two or three days; nor did she lose very much flesh or appetite. The cough continued to annoy her greatly; the expectoration increased, and the strength began to fail. She was first seen by him as an out-patient at the Infirmary on June 8, and on June 15 her case appeared to be so serious that she was placed under treatment in a private house.

There was almost incessant cough, preventing any sleep, except by the use of narcotics; large muco-purulent expectoration, which was very offensive; coarse *rales* over a large area of the centre of right lung, extending from the upper third of the scapula down to the ninth rib vertically, and from close to the spine to just above the nipple transversely. In front, there was great tenderness over the third, fourth, and fifth ribs, but no pointing or bulging of the intercostal spaces. Over the other parts of the lung respiration was free and normal, with the exception of some dullness at the base. The left lung was rather hyper-resonant. The paroxysms of coughing continued unceasingly for several hours at a time, almost abolishing sleep. There was not very much loss of flesh; the appetite was good; the pulse 110; temperature 102° F.

From the account given by a young friend who accompanied the patient to the dentist, it appeared very probable that a stump had escaped the operator, and had passed down the trachea into the right bronchus. This idea was confirmed by the friend stating that there was great searching of the mouth for a stump, and some alarm expressed. Further, she stated that before the patient came to the Infirmary, rings of cartilage, showing necrosis, were found in the sputum, which were horribly offensive. From June 15 to July 18, the case continued to increase in severity and urgency. The cough was almost incessant; bad bedsores formed from the continuous sitting posture; the temperature, however, only varied between 101.4° and 99°, and was often normal.

The pulse on June 16 was 146, on the 19, 160; then it fell gradually to 120 on the 26; again rose to 140 or more, and finally dropped, under large doses of brandy, to 120, where it remained until July 18, the thirty-third day of treatment, and five months from the date of the tooth drawing. During the whole of this time hectic fever continued to consume the patient's strength. The expectoration was putrid to the last degree, the room, and also the house itself, being only rendered bearable by chlorine gas freely used, with deodorants to the sputum. For some time past the sputum had contained a large quantity of broken down pulmonary tissue which settled in quantities to the bottom of the vessel. From two to three pints of this stinking stuff were expectorated daily. Occasionally it ran in a stream from the patient's mouth, and this caused a very tiresome aphthous condition. Large amphoric breathing with metallic tinkling was now heard in the centre of the lung over a space of over four inches square.

Having now, on July 18, five months from the access of cough, to deal with a gangrenous abscess of very large dimensions, with continuous destruction of lung tissue, which abscess had certainly existed for thirty-four days, if not longer, Dr. Stranger came to conclusion that nothing but puncture of the lung, reaching the abscess, and draining it, would give the patient any chance of surviving, even for a few days.

On July 18, the operation was performed. The needle of an aspirator was pushed quite home into the chest, just below the ninth rib, and at one inch behind the axillary line. A drop of pus appeared almost at once. Free vent was given to the pus, and a drainage tube inserted, the wound being dressed antiseptically. Recovery was slow, but by October 29, the patient had gained fourteen pounds in weight, and all signs of the immense abscess and its cavity had disappeared.

